# Supplementary material for: A neurotoxin that specifically targets Anopheles mosquitoes
Source: Nat Commun. 2019 Jun 28;10:2869. doi: 10.1038/s41467-019-10732-w (PMC6599013; doi:10.1038/s41467-019-10732-w)
Supplement: Supplementary file 1 — Supplementary Information [file 41467_2019_10732_MOESM1_ESM.pdf]

A neurotoxin that specifically targets Anopheles mosquitoes

Contreras et al.

Supplementary notes

A construct containing PMP1 gene in pHT 315 under *Bacillus thuringiensis* Cry3A promoter obtained from amplification of *Pbm* genomic sequence was first created and bioassayed with *A. aegypti* and *An. gambiae* larvae, along with constructs NTNH-PMP1 and NTNH-OrfX1-3-PMP1 (Fig 3A). Preliminary data showed no mortality. We next created an optimized construct with the *Bacillus thuringiensis* optimized sequence of PMP1 and cloned in pHT315 under a stronger *B. thuringiensis* promoter Cyt1A, but again, no toxicity was observed.

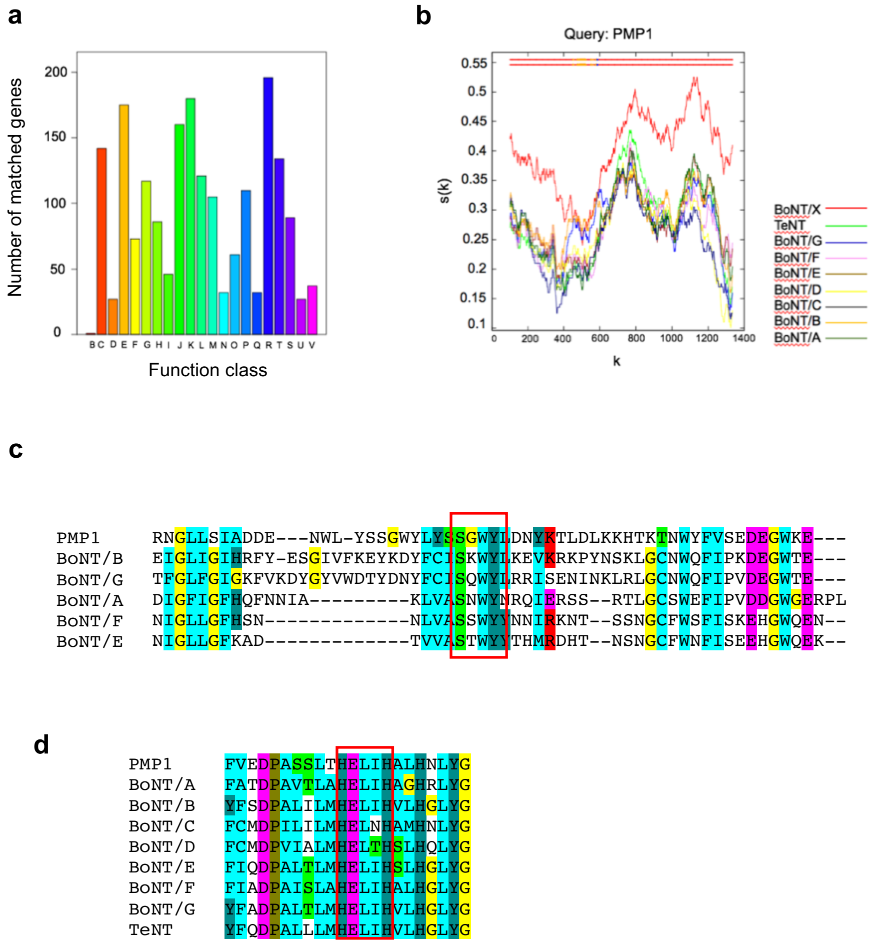

Supplementary Figure 1. *Pbm* gene functional annotation and analysis of PMP1 sequence. A) Gene functional annotation of *Pbm* genome. Annotated genes were aligned with COG function classification database. B: Chromatin structure and dynamics; C: Energy production and conversion; D: Cell cycle control, cell division, chromosome partitioning; E: Amino acid transport and metabolism; F: Nucleotide transport and metabolism; G: Carbohydrate transport and metabolism; H: Coenzyme transport and metabolism; I: Lipid transport and metabolism; J: Translation, ribosomal structure and biogenesis; K: Transcription; L: Replication, recombination and repair; M: Cell wall/membrane/envelope biogenesis; N: Cell motility; O: Posttranslational modification, protein turnover, chaperones; P: Inorganic ion transport and metabolism; Q: Secondary metabolites biosynthesis, transport and catabolism; R: General function prediction only; S: Unknown function; T: Signal transduction mechanism; U: Intracellular trafficking, secretion and vesicular transport; V: defense mechanisms. B) Distribution of the similarity between PMP1 and other CNTs. The X-axis represents the protein sequence position and the Y-axis shows the percentage of identity between PMP1 and other CNT in each sequence position. Alignment of the C terminus (C) and a LC fragment (D) of PMP1 and different BoNTs, with the SxWY ganglioside binding site and the conserved motif HELXH in the catalytic site respectively boxed.

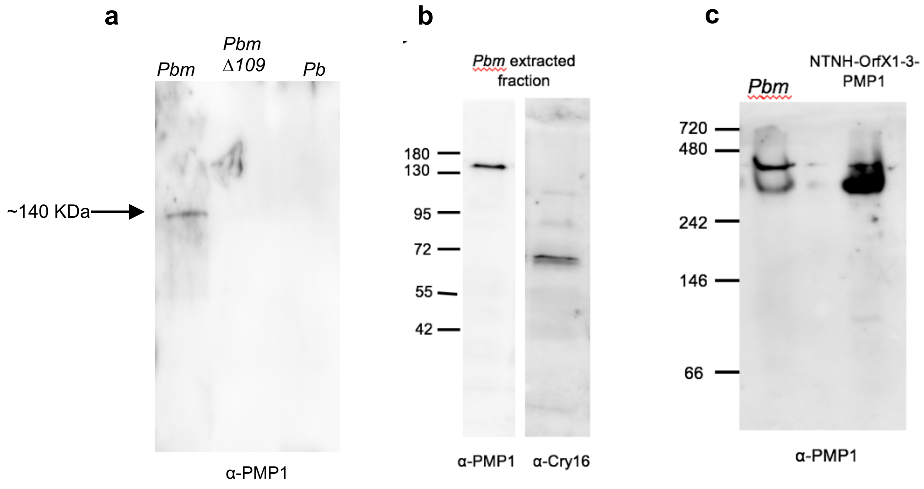

Supplementary Figure 2. Expression of proteins encoded by *ptox*. A) PMP1 is immunodetected in *Pbm* culture, but not in the *Pbm* loss of function mutant *Pbm*Δ*109*, or in the type strain *Pb*. B) Immunodetection of PMP1 and Cry16 in a western blot of the *Pbm* extracted fraction. C) Western blot of a native PAGE of Pbm extracted fraction (left lane) and whole culture of *B. thuringiensis* expressing NTNH-OrfX1-3-PMP1 construct (right lane). Complexes of similar sizes are observed in both samples.

a

MGSSHHHHHHSQDPMTKDRLAALQAAQSDDEDMPEDVAVPVEGSFMEDFFKEVEEIRMMIDKIQANVEEVKKKH
SAILSAPQSDEKTKQELEDLMADIKKTANRVRGKLKGIEQNIEQEEQQSKSNADLRIRKTQHSALSRKFVEVMT
EYNRTQTDYRERCKGRIQRQLEITGRATTNEELEEMLEQGNSAVFTQGIIMETQQAKQTLADIEARHADIIKLE
NSIRELHDMFMDMAMLVESQGEMIDRIEYHVE**HAMDYVQTATQDTKKALKYQSEQKLISE**ED**LEQKLISEEDL**

b

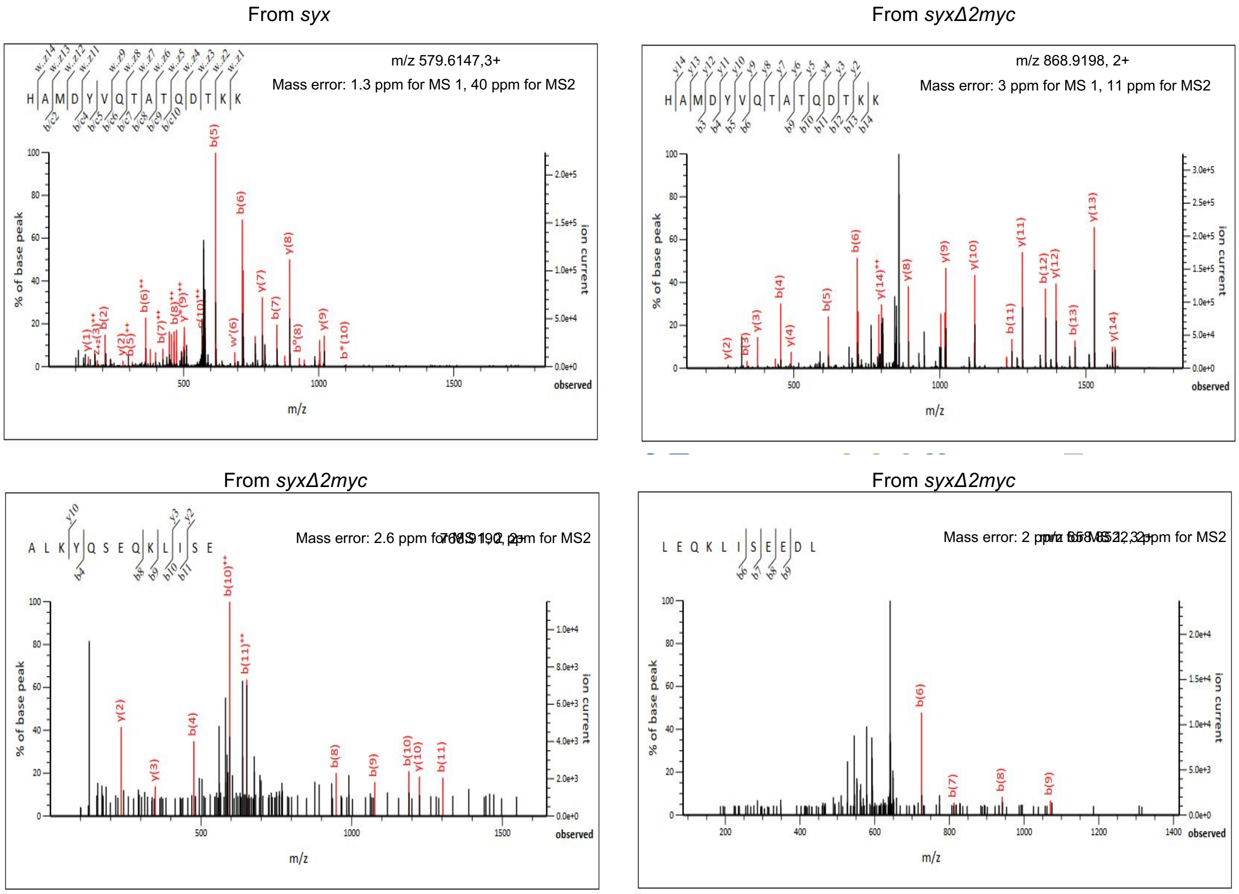

Supplementary Figure 3. Mass spectrometric data from syntaxin cleavage. A) Mass spectrometry of syxΔ2myc mutant cleaved with PMP1 LC identifies unique sequences (in blue) that are not observed in syxΔ2myc incubated with buffer or with the PMP1 E209Q mutant. B) Mass spectrometry spectra of the peptides from His-syntaxin detected only in the sample where His-syntaxin was incubated with PMP1 LC and not in the control or incubated with PMP1 E209Q mutant.

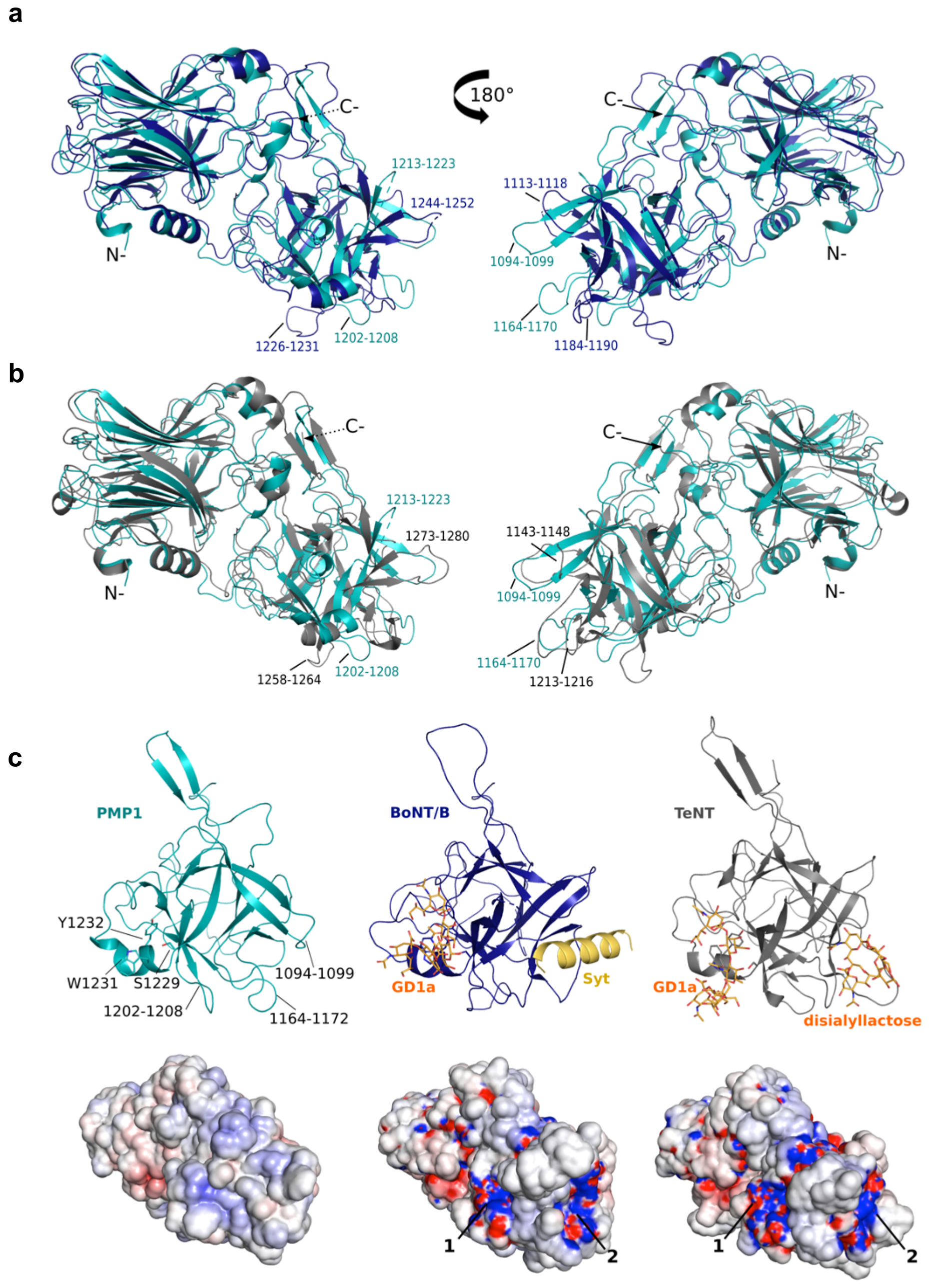

Supplementary Figure 4. Structure of PMP1-H_C. Superimposition of the PMP1-H_C crystal structure (cyan) with the crystal structure of BoNT/B-H_C (blue, PDB 4KBB) (A) and with the crystal structure of TeNT-H_C (grey, PDB 5N0B) (B). C) BoNT/B-H_C (blue, middle pannel) in complex with its receptors, synaptotagmin (yellow) and GD1a (orange) (PDB 4KBB) and TeNT (grey, right pannel) in complex with GD1a (orange) (PDB 5N0B) and a disialyllactose (orange, PDB 1YYN). The corresponding electrostatic surface potential of PMP1, BoNT/B and TeNT, is shown below each structure, calculated with the APBS tool in PyMol (scale -10 to +10, red to blue for the three structures). The receptor-binding sites of BoNT/B and TeNT are labeled as 1 (ganglioside) and 2 (protein receptor/sialic acid).

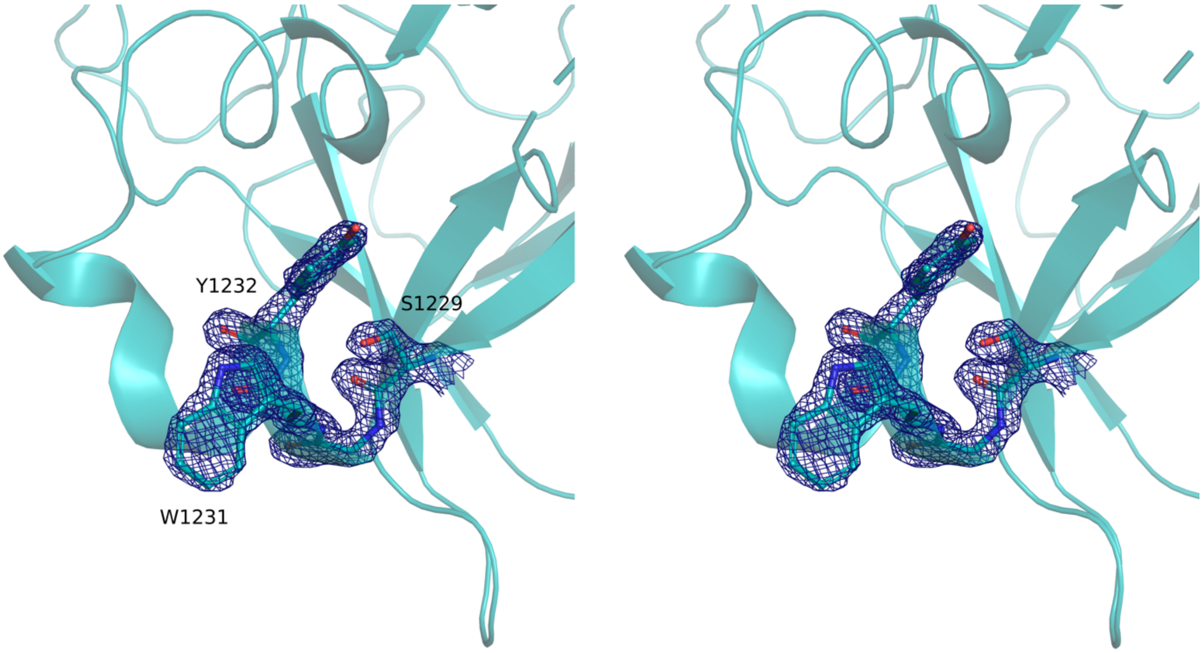

Supplementary Figure 5. Close-up view of the conserved SGWY motif of PMP1. Stereo image with ($2F_O$-F_C) electron-density map (contoured at 2.0 sigma) around residues [1229-1232].

Supplementary Table 1. Toxicity of *Pbm, Pbp* and *Bti* to 3rd instar *Aedes aegypti, Anopheles coluzzi* and *Anopheles stephensi* mosquito larvae and to *Drosophila melanogaster* larvae. LC50 is represented as volume of whole culture in 100 ml water and in CFU/ml water. Note that the lower toxicity of *Pbp* in comparison to *Pbm* could be at least partially due to a lower CFU/ml in the culture.

	**LC50 in µl culture (95% confidence interval) / in CFU per ml**			
	A. aegypti	*An. gambiae*	*An. stephensi*	*D. melanogaster*
Bti	$1.1x10^{-3}$ $(0.5x10^{-3}-2x10^{-3})$/ 902	$3.4x10^{-3}$ $(2.1x10^{-3}-4.9x10^{-3})$ /2788	$17.1x10^{-3}$ $(11.7x10^{-3}-24.8x10^{-3})$ /14022	ND
Pbm	$8x10^{-3}$ $(1.8x10^{-3}-22.5x10^{-3})$ / 45	$1.8x10^{-3}$ $(0.9x10^{-3}-3.2x10^{-3})$ /10	$3.9x10^{-3}$ $(2.7x10^{-3}-5.3x10^{-3})$ /22	ND/>$3.4x10^{6}$
Pbp	> 1000 / >400	$20.2x10^{-3}$ $(14.1x10^{-3}-28.5x10^{-3})$ / 1	ND	ND

Supplementary Table 2. Sequencing data of the *Pb malaysia* predicted genes.

Gene prediction	
Gene Number	3,835
Gene Length (bp)	3,319,296
GC Content in Gene Region (%)	28.77
Gene Length / Genome (%)	85.05
Gene Average Length (bp)	865
Intergenic Region Length (bp)	583.317
GC Content in Intergenic Region (%)	23.95
Intergenic Region Length /Genome (%)	14.94

Supplementary Table 3. Proteins identified by mass spectrometry from *Pbm* extracted fraction encoded by the 109, 7.2 and 4 kb *Pbm* and *Pbp* plasmids. Proteins from cry and Ptox toxin loci are highlighted in grey.

Protein	Mass	E1		E2	
		emPAI	score	emPAI	score
**109Kb plasmid**					
OrfX2	83955	18.50	9184	87.28	17648
Cry16	71353	9.04	4702	20.66	10002
Hemolysin 2	17452	44.17	4078	487.85	5926
Hemolysin 1	17189	17.06	1264	123.36	3497
OrfX3	54972	0.85	277	9.78	3397
NTNH	136370	1.54	2016	0.75	997
OrfX1	16828	4.59	301	18.10	1446
Cry17	71513	1.73	1435	0.06	50
CMP1	146228	0.95	1198		
P47	48060	0.19	203		
replication protein	46003	0.10	74	0.10	43
HA	51457	0.09	29		
unknown 003765	65930	0.07	22	0.07	25
transposase A	43000			0.10	23
unknown 003771	21473			0.21	23
unknown 003832	22289			0.21	22
chromosome partitioning parB	50615				
**7.2 Kb plasmid**					
unknown 002500	12266	0.40	51	ND	17
**4 Kb plasmid**					
unknown 002508	21658	0.21	189	0.21	119

Supplementary Table 4. Primers used for gene amplification, cloning and site-directed mutagenesis.

Primer	use	sequence
1	PMP operon Fw	GGCGCGCCATGGACATAATTGACAATGTAG
2	PMP operon Rv	CTCGAGCTATTCCTTCCATCCTTCATC
3	NTNH Fw	CCCGGGATCCAATAATAGAAGGATATCAAAT
4	NTNH Rv	GCGGCCGCCCATTCATCGAAACATTCCCATCAT
5	PMP1 Fw	CTCGAGATATTTATTATAGATACCTTAAAGG
6	PMP1 Rv	CCACTTAATTGGTCAAATAACTATTCTTAATATGCTA
7	E209Q Fw nested	CGGCATCGAGCCTGACGCACCAACTGATCCATGCTCTGCAC
8	E209Q Rv nested	GTGCAGAGCATGGATCAGTTGGTGCGTCAGGCTCGATGCCG
9	PMP1/E209Q Fw	GGATCC CTGCAAATCCGTGTCTTTAACTATAACG
10	PMP1/E209Q Rv	GGGCCCACATACGGGATAATCCAAGAGATGTC
11	PMP1 Hc Fw	GGATCCGAATGCCCTGATCGATCGCCTGGGTA
12	PMP1 Hc Rv	AAGCTTTCATTCTTTCCAACCTTCATCTTCC
13	PMP1 LC Fw	CCATGGACTACAAAGACGATGACGACAAGCTGCAAATCCGTGTCTTTAACTATAACG
14	PMP1 LC Rv	AAGCTTTCACAGTTTAACTTTTTTCGAGATCAG
15	His syx Fw	CGGGATCCGATGACGAAGGACAGATTAGCAGCCCT
16	His syx Rv	GGCGCGCCTTACAGGTCTTCTTCAGAG
17	H252N Fw	GATTGATCGTATAGAATATAACGTCGAACATGCAATGG
18	H252N Rv	CCATTGCATGTTCGACGTTATATTCTATACGATCAATC
19	L271V Fw	CAAGACACAAAGAAAGCGGTCAAATATCAAAGCAAAGC
20	L271V Rv	GCTTTGCTTTGATATTTGACCGCTTTCTTTGTGTCTTG
21	T264V Q265S Fw	GATTATGTTCAAACAGCGGTGTCTGACACAAAGAAAGCGC
22	T264V Q265S Rv	GCGCTTTCTTTGTGTCAGACACCGCTGTTTGAACATAATC
23	Q261E T262R Fw	CAATGGATTATGTTGAAAGAGCGACACAAGACACAAAG
24	Q261E T262R Rv	CTTTGTGTCTTGTGTCGCTCTTTCAACATAATCCATTG
25	M257V Fw	CACGTCGAACATGCAGTGGATTATGTTCAAACAGCGAC
26	M257V Rv	GTCGCTGTTTGAACATAATCCACTGCATGTTCGACGTG
27	syx Δ2myc 1	GTTCCAGGTCTTCTTCAGAGATCAGTTTCTGTTCGCTTTGATATTTAAGCGCTTTCTTTG
28	syx Δ2myc 2	GGCGCGCCTTACAGGTCTTCTTCAGAGATCAGTTTCTGTTCCAGGTCTTCTTCAGAGATCAG
