# Supplementary material for: A neurotoxin that specifically targets Anopheles mosquitoes
Source: Nat Commun. 2019 Jun 28;10:2869. doi: 10.1038/s41467-019-10732-w (PMC6599013; doi:10.1038/s41467-019-10732-w)
Supplement: Supplementary file 3 — Reporting Summary [file 41467_2019_10732_MOESM3_ESM.pdf]

nature research

Corresponding author(s): Sarjeet Gill

Last updated by author(s): May 11, 2019

Reporting Summary

Nature Research wishes to improve the reproducibility of the work that we publish. This form provides structure for consistency and transparency in reporting. For further information on Nature Research policies, see Authors & Referees and the Editorial Policy Checklist .

Statistics

For all statistical analyses, confirm that the following items are present in the figure legend, table legend, main text, or Methods section.

n/a	Confirmed	
☐	☒	The exact sample size (n) for each experimental group/condition, given as a discrete number and unit of measurement
☐	☒	A statement on whether measurements were taken from distinct samples or whether the same sample was measured repeatedly
☐	☒	The statistical test(s) used AND whether they are one- or two-sided<br>*Only common tests should be described solely by name; describe more complex techniques in the Methods section.*
☐	☒	A description of all covariates tested
☐	☒	A description of any assumptions or corrections, such as tests of normality and adjustment for multiple comparisons
☐	☒	A full description of the statistical parameters including central tendency (e.g. means) or other basic estimates (e.g. regression coefficient) AND variation (e.g. standard deviation) or associated estimates of uncertainty (e.g. confidence intervals)
☐	☒	For null hypothesis testing, the test statistic (e.g. F, t, r) with confidence intervals, effect sizes, degrees of freedom and P value noted<br>*Give P values as exact values whenever suitable.*
☒	☐	For Bayesian analysis, information on the choice of priors and Markov chain Monte Carlo settings
☐	☒	For hierarchical and complex designs, identification of the appropriate level for tests and full reporting of outcomes
☒	☐	Estimates of effect sizes (e.g. Cohen's d, Pearson's r), indicating how they were calculated

Our web collection on statistics for biologists contains articles on many of the points above.

Software and code

Policy information about availability of computer code

Data collection	No previously unreported custom computer code or algorithm was used
Data analysis	Data was analyzed with Excel and plotted using Origin software.

For manuscripts utilizing custom algorithms or software that are central to the research but not yet described in published literature, software must be made available to editors/reviewers. We strongly encourage code deposition in a community repository (e.g. GitHub). See the Nature Research guidelines for submitting code & software for further information.

Data

Policy information about availability of data

All manuscripts must include a data availability statement. This statement should provide the following information, where applicable:

- Accession codes, unique identifiers, or web links for publicly available datasets
- A list of figures that have associated raw data
- A description of any restrictions on data availability

The atomic coordinates and structure factors (code 6HOX) have been deposited in the Protein Data Bank. Accession numbers of CP032452, CP032455 for Pbm genome and pPbmMP, respectively are available at the NCBI database. The Pbp and Pb whole genome shotgun projects have been deposited at DDBJ/ENA/GenBank under the accession numbers RAHB00000000 and QZNC00000000, respectively. Data will be available on publication of this manuscript.

Field-specific reporting

Please select the one below that is the best fit for your research. If you are not sure, read the appropriate sections before making your selection.

Life sciences study design

All studies must disclose on these points even when the disclosure is negative.

Sample size	No sample-size calculation was performed. Sample size was selected to detect differences between groups.
Data exclusions	Data was only excluded for failed experiments, which included where the tested protein was inactive due to degradation.
Replication	Replicate experiments were successful.
Randomization	Mosquito larvae were randomly chosen for bioassay from synchronized egg hatch.
Blinding	Mosquito larvae and mice used for experiments were from the same strains and genotypes, so investigators were not blinded for animal selection.

Reporting for specific materials, systems and methods

We require information from authors about some types of materials, experimental systems and methods used in many studies. Here, indicate whether each material, system or method listed is relevant to your study. If you are not sure if a list item applies to your research, read the appropriate section before selecting a response.

Materials & experimental systems

n/a	Involved in the study
☐	☒ Antibodies
☒	☐ Eukaryotic cell lines
☒	☐ Palaeontology
☐	☒ Animals and other organisms
☒	☐ Human research participants
☒	☐ Clinical data

Methods

n/a	Involved in the study
☒	☐ ChIP-seq
☒	☐ Flow cytometry
☒	☐ MRI-based neuroimaging

Antibodies

Antibodies used	Rabbit antibodies against the PMP1 peptide in the heavy chain (GFENIDFSEPEIRY) was produced through Genscript . GST tag antibody was purchased from GE Healthcare, His tag antibody was from Genscript, Drosophila syntaxin antibody was from Developmental Studies Hybridoma Bank, University of Iowa and myc tag antibody from Cell Signaling.
Validation	Proteins produced in the study detected by GST tag, His tag and myc tag commercial antibodies contain exactly the same sequence as the one described in the corresponding vendors web pages.<br>Drosophila polyclonal syntaxin antibody was used for Anopheles syntaxin detection. Syntaxins of both dipteran are highly homologous.<br>PMP1 antibody was detected in Pbm culture but not in the mutant Pbm109, proving its specificity. It also detects expressed PMP1

Animals and other organisms

Policy information about studies involving animals; ARRIVE guidelines recommended for reporting animal research

Laboratory animals	Aedes aegypti Orlando strain, Anopheles coluzzi, Swiss Webster female mice
Wild animals	*Provide details on animals observed in or captured in the field; report species, sex and age where possible. Describe how animals were caught and transported and what happened to captive animals after the study (if killed, explain why and describe method; if released, say where and when) OR state that the study did not involve wild animals.*
Field-collected samples	*For laboratory work with field-collected samples, describe all relevant parameters such as housing, maintenance, temperature, photoperiod and end-of-experiment protocol OR state that the study did not involve samples collected from the field.*
Ethics oversight	Animal use was approved by the University of California, Riverside Institutional Animal Care and Use Committee.

Note that full information on the approval of the study protocol must also be provided in the manuscript.
