# Supplementary material for: A neurotoxin that specifically targets Anopheles mosquitoes
Source: Nat Commun. 2019 Jun 28;10:2869. doi: 10.1038/s41467-019-10732-w (PMC6599013; doi:10.1038/s41467-019-10732-w)
Supplement: Supplementary file 4 — Description of Additional Supplementary Files [file 41467_2019_10732_MOESM4_ESM.pdf]

Description of Additional Supplementary Files

File Name: Supplementary Data 1
Description: Annotation of Pbm and Pbp 109Kb plasmid

File Name: Supplementary Movie 1
Description: PMP1 produces slowing of motion in Aedes mosquito larvae. Fragments of video recording (3 s) of 15 A. aegypti 4th instar larvae after 15 min and 1 to 6h after injection with water (control), PMP1 and PMP1 E209Q mutant.
